# LONELINESS AMONG LONG-TERM SPOUSAL CAREGIVERS: A GENDER-BASED ANALYSIS USING THE CLSA

**DOI:** 10.1093/geroni/igae098.0101

**Published:** 2024-12-31

**Authors:** Lun Li, Andrew Wister, Yeonjung Lee

**Affiliations:** MacEwan University, Edmonton, Alberta, Canada; Simon Fraser University, Vancouver, British Columbia, Canada; Chung-Ang University

## Abstract

Spousal caregivers tend to undertake the most care for their loved ones. As a result, spousal caregivers also experience worse caregiving outcomes, including loneliness, than other types of caregivers. This study used three waves of data from the Canadian Longitudinal Study on Aging (2011 to 2021), and longitudinal analyses with the Linear mixed model were performed to examine the loneliness (measured by UCLA 3-item loneliness scale) of spousal caregivers over time. A total of 1569 participants were identified as long-term spousal caregivers (849 male and 720 female). The results showed that female spousal caregivers reported both a higher level of loneliness at the beginning and a greater increase of loneliness over time than male spousal caregivers. Besides participants’ demographic, social-economic and health-related factors, caregiving hours, social participation, and social support are the key predictors of loneliness. At the same time, female spousal caregivers experience a steeper increase in caregiver hours, and a greater decrease in social participation and social support over time. These disparities in changes over time amplify the negative impacts of caregiving on female spousal caregivers. The findings reveal the greater caregiver burden taken by female spousal caregivers than male ones over time. The study supports future programs and services for female spousal caregivers to manage caregiving tasks better and maintain active social interaction to balance caregiving and social life.

